# Anticipatory prescribing of injectable controlled drugs (ICDs) in care homes: a qualitative observational study of staff role, uncertain dying and hospital transfer at the end-of-life

**DOI:** 10.1186/s12877-024-04801-z

**Published:** 2024-04-03

**Authors:** Diana Teggi, Kate Woodthorpe

**Affiliations:** Department of Social and Policy Sciences, Centre for Death and Society (CDAS), University of Bath, Bath, UK

**Keywords:** Anticipatory prescribing, Care home palliative care, End-of-life care, Older adults, Nursing home

## Abstract

**Background:**

The anticipatory prescribing of injectable controlled drugs (ICDs) by general practitioners (GPs) to care home residents is common practice and is believed to reduce emergency hospital transfers at the end-of-life. However, evidence about the process of ICD prescribing and how it affects residents’ hospital transfer is limited. The study examined how care home nurses and senior carers (senior staff) describe their role in ICDs prescribing and identify that role to affect residents’ hospital transfers at the end-of-life.

**Methods:**

1,440 h of participant observation in five care homes in England between May 2019 and March 2020. Semi-structured interviews with a range of staff. Interviews (*n* = 25) and fieldnotes (2,761 handwritten A5 pages) were analysed thematically.

**Results:**

Senior staff request GPs to prescribe ICDs ahead of residents’ expected death and review prescribed ICDs for as long as residents survive. Senior staff use this mechanism to ascertain the clinical appropriateness of withholding potentially life-extending emergency care (which usually led to hospital transfer) and demonstrate safe care provision to GPs certifying the medical cause of death. This enables senior staff to facilitate a care home death for residents experiencing uncertain dying trajectories.

**Conclusion:**

Senior staff use GPs’ prescriptions and reviews of ICDs to pre-empt hospital transfers at the end-of-life. Policy should indicate a clear timeframe for ICD review to make hospital transfer avoidance less reliant on trust between senior staff and GPs. The timeframe should match the period before death allowing GPs to certify death without triggering a Coroner’s referral.

## Introduction

Older adults, their carers, and clinicians may experience significant anguish as a result of poorly managed symptoms in the last days of life [1–4]. The prescription of injectable controlled drugs (ICDs) ahead of potential need has become a key element of community end-of-life care provision in the UK, Australia and New Zealand [5–9]. ICDs include several injectable drugs controlled under national misuses of drugs legislation [5–8, 10, 11]. In the UK, where this study is set, ICDs typically are Schedule 2 and 3 injectable drugs [12, 13] such as morphine, oxycodone, fentanyl or diamorphine (for pain) and midazolam (for agitation) [5, 14–16]. The practice of anticipatory ICDs prescribing is based on healthcare professionals’ belief that the presence of ICDs in the patient’s residence reassures the patient and their carers, provides rapid symptom relief, and prevents emergency hospital admission [4, 17–19]. However, evidence supporting this is limited, of mixed quality, and mostly based on general practitioners’ (GPs) and district nurses’ experience of ICDs prescribing and administration in the home setting [5–8, 10, 11, 14, 19]. Moreover, the evidence base supporting anticipatory prescribing in the care home setting, where care home nurses and senior carers (senior staff) control older adults’ access to GPs and district nurses, and thus ICDs prescribing and administration, is particularly poor [14]. As the main prescribers of ICDs to care home residents [15, 20], evidence suggests that GPs prescribe ICDs in about half of residents’ deaths, and weeks, months or even years ahead of death [15, 20]. Yet how care home staff define their role in guiding ICD prescription and using the on-site availability of ICDs for a named resident, and consider that role to affect residents’ hospital transfers at the end of life, is not well-understood. The few studies linking anticipatory prescribing to a reduction in care home residents’ hospital transfers evaluate the outcome of single interventions [17, 18], failing to investigate the care home staff’s common practices which may hinder or facilitate this reduction. This is a key gap in knowledge considering that care home senior staff are responsible for triggering residents’ hospital transfers through emergency services [21], residents’ dying is extremely difficult to predict [22], and most hospital admissions of care home residents are inappropriate at the end-of-life, leading to traumatic transfers and poor symptom control [16, 23–25].

The aim of this study is to explore how senior staff describe their role in ICD prescribing in care homes, identify that role to affect residents’ transfers to hospital at the end-of-life, and provide recommendations for future ICD review practice. This study is based on observed practice and staff interviews in English care homes, where anticipatory prescribing is common practice [14] and residents experience a high rate of hospital admission in the last month of life (34%) [26] and death (28.5%) [27]. Non-emergency research in this area is thus needed and qualitative observational studies of anticipatory prescribing in care homes such as this are rare [14]. Of the few that do exist, they largely exclude care homes without nurses and neglect senior staff’s role and agency in guiding GPs to prescribe ICDs and managing their availability on the care home’s premises [15].

## Methods

### Design

Data for this study originates from a qualitative observational study of end-of-life care (EOLC) practice, combining participant observation and staff interviews in five English care homes [28]. The study adopted a social constructivist new-materialist ontology and epistemology [29], and an inductive approach to data collection [28, 30]. New materialism defines causality as an objective relation through which “*one event produces another event*” [31] and posits that how people make sense of such relations contributes to their production [29]. In line with the new-materialist paradigm, qualitative research methods which allowed interaction with participants and the co-construction of interpretative hypotheses were chosen. Data collection occurred in two phases in each care home. The first phase involved DT, a medical sociologist, conducting participant observation of staff’s EOLC practices. The second phase involved DT conducting semi-structured interviews with staff. This approach allowed the hypothesis that staff considered their use of ICDs prescribing to prevent hospital transfers at the end-of-life to emerge inductively from observation of staff’s practice. The hypothesis was then explored in interviews with staff by asking open-ended questions about the process and outcomes of anticipatory prescribing. This approach reduced confirmation bias and allowed for unexpected observations to feed into themes development.

The University of Bath Social Science Research Ethics Committee (SSREC) approved this study (reference number S18-010). Staff gave their written informed consent to be observed and interviewed. Residents gave their verbal informed consent to be observed. No residents without mental capacity to consent were observed.

### Recruitment and participants

Care homes were recruited by DT through a gatekeeper or by contacting the manger directly. Purposive sampling was used to select five sites in the South-West of England providing a mix of nursing and residential services, varying from medium to large size, and belonging to one national charitable provider, one local for-profit provider, and one single-owner provider (Table 1). DT approached staff to seek consent for observation and interviewing. The observation sample included only staff having daily interactions with residents, namely carers, senior carers and nurses (Table 2). The interview sample included 25 staff working across the five care homes, comprising five managers, ten carers, six nurses and four senior carers (Table 2). The weight given to social care staff (carers and senior cares), nursing staff (nurses) and managerial staff (managers) reflects the composition of care home staff’s roles in England [32]. Senior staff roles (nurses in nursing settings and senior carers in residential settings) were oversampled with respect to ancillary roles (carers) because senior staff are responsible for liaising with GPs, district nurses and emergency services [21]. In the UK, any doctor or nurse prescriber can prescribe ICDs to a patient expected to die out of hospital. An ICD prescription is valid for 28 days from the date on the prescription [13]. Further, once delivered by the pharmacy, any registered nurse or doctor can administer ICDs for as long as they are in-date [13]. GPs and district nurses deliver out-of-hospital care in the UK. GPs and district nurse prescribers are responsible for liaising with care home nurses and senior carers to prescribe ICDs in nursing and residential settings respectively [15, 19]. District nurses are responsible for liaising with senior carers to administer ICDs in residential settings [20]. Whereas care home nurses are responsible for administering ICDs in nursing settings [15].

**Table 1:** Characteristics of the care homes’ sample

Care Home	Services	Number of Beds	Provider Type	Provider Size
1	Residential general	33 (small size)	Not-for-profit	National
2	1st floor: residential dementia (32 beds) 2nd floor: nursing general (33 beds) 3rd floor: residential semi-independent living apartments (10 beds)	75 (large size)	Not-for-profit	National
3	Nursing general	65 (large size)	Not-for-profit	National
4	1st floor: residential dementia (20 beds) 2nd to 4th floor: nursing general and nursing dementia (45 beds)	65 (large size)	For-profit	Local
5	Nursing dementia	51 (medium size)	For-profit	Single-home owner

**Table 2:** Characteristics of the staff’s sample

Position		Role & Hierarchy	Number Observed	Number Interviewed
	Manager	There is only one manager in each care home. Managers are responsible for the management of all staff, and legally responsible for residents’ safety.	N.A.	5
Senior staff	Nurse	Nurses are the senior staff in nursing homes (or floors). They are accountable to the manager and responsible for the management of part of the nursing home and residents. This typically includes 3–4 carers and 30–35 residents. Nurses are responsible for administering medication (ICDs included) and liaising with the GP and the relatives, close companions, or Lasting Powers of Attorneys (LPAs) of the residents.	10	6
Senior staff	Senior carer	Senior carers are the senior staff in residential homes (or floors). They are accountable to the manager and responsible for the management of part of the residential home and residents. This typically includes 3–4 carers and 30–35 residents. Senior carers are responsible for administering all medication except ICDs which require a registered nurse (typically a district nurse) for administration. Senior carers are responsible for liaising with the GP, district nurses and the relatives, close companions, or LPAs of the residents.	9	4
	Carer	Carers are accountable to the manager and the nurse or senior carer allocated to their floor or part of the care home. Carers are responsible for helping residents complete their activities of daily living, such as getting dressed and washed, use the toilet, change their nappies, eat and hydrate. Carers are not responsible for administering medication to the residents nor liaising with the GPs, district nurses and the relatives, close companions, or LPAs of the residents.	35	10

### Data collection

DT performed approximately 1,440 h of observation shadowing 35 carers and 19 senior carers across the five care homes between May 2019 and March 2020. Shadowing consisted in following staff as they went about their daily routine and interactions with residents for the duration of a 12-hour day or night shift, three or four times a week. DT’s observations focussed on the identification and care of residents expected to die. DT took fieldnotes during breaks and at the end of each observation day. This generated 2,761 pages of handwritten fieldnotes in eleven A5 notebooks. DT interviewed staff at the end of the observation period in each care home to ask follow-up questions on anticipatory prescribing. Semi-structured interviews lasted between 40 and 120 min.

### Data analysis

Fieldnotes were digitised and the published excerpts pseudonymised by DT. Interviews were professionally transcribed into intelligent verbatim and pseudonymised by DT. DT and KW analysed the fieldnotes and interview transcripts using ATLAS.ti, a software for qualitative data-analysis. The authors applied reflexive thematic-analysis, a data-driven interpretative approach positing a recursive relationship between inductive data-analysis and deductive themes-development [33, 34]. DT conducted the first coding round inductively, identifying any instance of anticipatory prescribing by a GP. This operation identified 124 data excerpts across fieldnotes and interview transcripts. In the second coding round, DT searched the first 60 data excerpts for patterns of shared meaning (themes) around staff’s involvement in ICDs prescribing and their use of prescribed ICDs. KW coded these first 60 data excerpts independently. The authors compared the initial themes and refined them through debate and the triangulation of fieldnotes and interview data for each care home. This process delivered two subthemes explaining senior staff’s role in the GP prescription of ICDs, and three subthemes explaining senior staff’s use of prescribed ICDs. In the third coding round, DT applied the five subthemes deductively to code the complete dataset of 124 data excerpts. KW reviewed 10 data excerpts for each subtheme and reflected on interpretative differences with DT. This defined the boundaries and relevance of the five final subthemes.

## Results

### Senior Staff’s Role in Anticipatory Prescribing, Storing and Reviewing

This section describes the role of care home senior staff in the process of ICDs prescribing, storing and review by the resident’s GP.

#### Senior staff initiate most anticipatory prescribing

Of the ICDs prescriptions observed, most were performed by the GP on the advice of care home senior staff. Staff reported that they felt that most ICD requests were initiated by them:

*“there’s a time we’d say [nurses], ‘I think this person is entering end-of-life.’ If the doctor [GP] agrees, and they would say, ‘No, you know your residents better than me, what do you think?’ That’s when the doctor will prescribe anticipatory medications.” [Nurse 3]*.

The context for these conversations between senior staff and GPs were the GP weekly rounds. Typically, these involved a GP (from the GP practice allocated to the care home) visiting five to six residents selected by senior staff among the $\sim 30$ residents usually living on a care home floor (Table 1). Rounds lasted 30 to 50 min in total. These rounds constituted the principal mechanism for senior staff to influence a GP’s decision to prescribe ICDs by briefing the GP about a resident’s worsening condition and/or selecting a resident for consecutive GP rounds as their health declined.

Senior staff sought to anticipate residents’ deaths and invite GPs to prescribe ICDs as residents were *“entering the end-of-life”*, namely before they might *“become symptomatic”* and need ICDs administered *[nurse 3]*. This reflected the time needed to source the ICDs, which was a laborious process taking up to 24 h (Table 3), and the difficulty of accurately predicting residents’ specific time of death. To forecast a resident’s death and propose them for ICDs, senior staff utilised the key stages of deterioration described in Table 4. These local heuristic criteria identify tipping points at which death might occur rapidly due to the accumulation of multiple symptoms and/or events indicating significant deterioration.

**Table 3:** Prescribing and sourcing ICDs in the care home context

The steps	Senior staff experience
Step 1: Contact the GP to prescribe the ICDs. Out-of-hours GPs are more reluctant than the resident’s GP to prescribe ICDs because they are not familiar with the resident’s medical history.	*“if a resident is end of life and we need those medications urgently then it’s very frustrating [because] we need to try in time to quickly get the meds in, so we need to wait for the GP to do the prescription” [Senior carer 1]* *“the out of hours GPs, I shouldn’t say they’re afraid, [but] they’re more reluctant to prescribe end of life medications for a person they don’t know. So what they will do, let’s say we call them out for somebody, they might say […] “Well, it’s Sunday today, you can call your GP to come in tomorrow, let’s try some Oramorph liquid, let’s do that”. Which sounds quite appropriate because just think about yourself going in somewhere, seeing someone the first time” [Nurse 4]*
Step 2: Order the ICDs through the pharmacy. There are some caveats which complicate the ordering of ICDs through the pharmacy: a) only selected pharmacies store ICDs, b) surgeries cannot fax controlled drugs prescriptions, and c) not all surgeries have electronic prescription services.	*“[Senior carer 5] complained that morphine and midazolam are almost impossible to get out-of-hours (during the weekend or at night) because they are controlled drugs and only a few pharmacies store them.” [Fieldnote extract 13:277]* *“we can’t use other pharmacies but our pharmacy that we work with […] and because it’s a controlled drug prescription the surgery can’t fax it to them [pharmacy] so somebody from [pharmacy] have to go and collect it” [Senior carer 1]*
Step 3: The pharmacy can take up to 24 h to deliver the ICDs. Senior staff feel the need to chase the pharmacy when ICDs are needed urgently, even when these were prescribed electronically.	*“the doctor will prescribe it electronically, it will go straight to [local] pharmacy [with which the Home works] so after the GP visit we have to inform the [local] pharmacy to […] keep an eye because we need it tonight, will you please deliver it tonight?” [Nurse 6]* *“If you don’t have the meds, send somebody over or chase [pharmacy] and see, when are they going to be here because we need them as soon as possible.” [Senior carer 2]*
Step 4: Collect the administration authorisation chart from the GP surgery. ICDs cannot be administered without the chart stating the dosage. GPs complete the chart at the surgery. Senior staff need to free a member of staff to collect the Chart from the surgery. This is difficult due to high care workloads.	*“the GP does that chart [administration authorisation chart] at the surgery and he will usually leave it there and then it’s up to us to coordinate. […] Sometimes the [district] nurses, kindly, if they’re around the area will go and collect the chart from the surgery but these new nurses don’t really do that so then […] I need to find somebody, a member of staff, a volunteer, anyone, to go to surgery to collect that chart because without that chart, they [district nurses] can’t give the person any medication” [Senior carer 1]* *“[Nurse 7] asked me to go the GP practice and collect the Palliative Care Chart [administration authorisation chart] for resident [Ellen] because all carers were busy with residents and he could not leave the floor. The practice was only a 5-minute walk from the nursing home.” [Fieldnote extracts 13:25–26]*

**Table 4:** Key stages of deterioration (tipping points) triggering ICDs prescription

Stage description	Example
The stages are ordered according to an ideal dying trajectory. In practice, not all residents go through all stages in the described order. Most residents go through only some of the stages, and they do so at very different paces, varying from hours, to days, weeks, months or even years. Senior staff does not consider each stage in isolation, but as a significant step in the overall process of deterioration.	
Stage 1: Recurrent hospital admissions in a few months with the same symptoms (usually up to three)	*“It’s events building up to that time. If somebody’s had multiple hospital admissions, they’ve come out of hospital, they’ve been on antibiotics, as soon as the antibiotics stop they get another chest infection, back into hospital, invasive treatments– injections, ivs [intravenous injections]. We usually say two or three times with the same sort of problem, same symptoms, somebody seems as though they’re deteriorating. So when it’s sort of the whole package you then think […] that any further hospital admissions is really not quality of life, not beneficial, not doing what you would expect it to do. Now we’re at the time where we need to be thinking of […] palliative care and end-of-life care.” [Nurse 2]*
Stage 2: Hospital doctors advise against future hospital admission, often by discharging the resident with a not-for-readmission letter or ReSPECT form.	*“When resident [Albert] was discharged mid-November with an updated ReSPECT form insisting on no future hospital admission, he looked much frailer than before and [nurse 9] called in the GP to prescribe end-of-life medication [ICDs] on the same day” [Fieldnote extract 15:214]*
Stage 3: Multiple courses of antibiotics (usually up to three) are ineffective to clear a major infection (usually a chest infection).	*“The GP was in today and he visited resident [Ivan]. His chest infection has cleared, apparently. [Nurse 5] commented that otherwise the GP would have not prescribed a fourth round of antibiotics but end of life medication instead. This is what they usually do [GPs] when antibiotics stop working.” [Fieldnote extract 17:28]*
Stage 4: Steep decline in alertness, mobility, and appetite levels, culminating in the resident lying in bed in a deeply sleepy or comatose state, unable to eat and drink.	*“[Jill] [resident with Alzheimer’s disease] was reviewed [visited] for end of life meds [ICDs], because she was deteriorating, but the GP said, “She’s not quite there yet”. And I agreed, she was standing up, she was smiling. Since the last five days she’s not eating, not smiling, she’s just very much gone and she is at that brink of she could suddenly rapidly deteriorate and that would be it. So we need to get them in place today […] she’s on the GP list because she’s now on that cusp and she’s not gonna recover, but she could go suddenly quickly.” [Senior carer 3]*

#### Senior staff encourage GPs not to de-prescribe but review anticipatory medications instead

Death was not always certain. Many residents survived one or more key stages of deterioration (Table 4) from which senior staff and GPs expected them to die and for which ICDs had been prescribed, as this manager noted:

*“A lady lived here 13 years and on four or five occasions we thought she was going to pass away, and she pulls through and she picks up again”. [Manager 3]*

The combined difficulties of predicting the time of death and sourcing ICDs at short notice (Table 4) led senior staff to ask GPs to *not* de-prescribe ICDs when a resident stabilised. ICDs were thus stored in the care home for as long as residents survived, ranging from a few days or weeks to *“three, four, five, [or] six months” [manager 2]* or even *“two or three years” [nurse 1].* Storing ICDs allowed senior staff to manage uncertainty about a residents’ dying, as this senior carer explained:

*“We can store end-of-life medication [ICDs] for years, until it expires, you know? But we’ve got it in the building because we’ve asked the GP– we’ve requested […] Then, what we do is they [residents] go on to palliative care with the district nurses and say, “Right, we’ve got palliative care medication [ICDs] in place, they are deteriorating, they don’t quite need it yet”. [Senior carer 3]*

Senior staff usually did not seek additional GP input about whether and when a dying resident needed ICDs administered. Care home nurses relied on their own professional judgement, while senior carers relied on district nurses’ professional judgement. However, both care home nurses and senior cares requested GPs to review the clinical appropriateness of *the option* to administer ICDs, namely ICDs prescriptions and administration charts, when storing ICDs for many months or years, as another senior carer explained:

*“[June] has bounced back God knows how many times and we’ve got the medication [ICDs] that’s been there for years, she keeps going– but we keep it. She’s not on regular palliative care checks because they know [GPs] that when that time comes we’ll call them” [Senior carer 2]*.

ICDs can be administered by a registered nurse or doctor for as long as they are not expired once delivered by the pharmacy [13]. Nonetheless, care home nurses and senior carers invited GPs to review ICDs to make sure that care home and district nurses felt comfortable to administer them months or years after prescription. This indicates that care home nurses and senior carers perceived, respectively, their own and district nurses’ professional autonomy about ICD administration to depend on the time elapsed since the GP last reviewed the ICDs. There was however ambiguity concerning the length of this time.

While national clinical guidelines advise prescribers to review ICDs prescriptions and administration charts [35], they do not specify a timeframe for review. Equally, local clinical guidelines in the areas of this study did not provide a timeframe for ICD revision. Senior staff navigated this grey area by inviting the GP to visit residents with ICDs ad-hoc (as their health deteriorated) or periodically on the GP weekly rounds. Both strategies depended on and contributed to building trusting relationships with the GPs, as the senior carer above and this nurse and manager explained:

*“if we [nurses] get a feeling [that a resident with ICDs might be dying], we have to call the doctor in, regardless. Because, if we’ve got someone who is end-of-life care [with ICDs] and we don’t have a GP visit within a couple of weeks, they will go to the Coroner if they pass away.” [Nurse 5]*.

*“So they [GPs] trust us, they’ll come in and do the certification [of medical cause of death] […] if they felt that there was anything untoward they would then say stop we’ve got to get the Coroner in […] but if residents have anticipatory medications [ICDs] we would then make sure they are checked regularly so they don’t have to go down the Coroner route and the doctor knows what’s going on.” [Manager 1]*.

In the senior staff’s experience, the perceived need to review the ICDs clinically overlapped with the desire to avoid a Coroner’s investigation legally. Crucially, both ICDs reviews and Coroner’s investigations hinged on the GP’s authority. A GP has a legal obligation to refer a death to the Coroner when they cannot certify the medical cause of death confidently [36], suspect neglect, or have failed to visit the resident within 28 days of death [37] (14 days pre-pandemic) [38]. Coroner’s referrals complicate relatives’ access to the body and delay funerals significantly. They are however unlikely to involve in-depth investigation when the GP releases the medical certificate of cause of death (MCCD) and believes safe care was provided [39]. By contrast, when the GP refuses to release the MCCD or reports neglect, the Coroner typically leads a detailed investigation to establish the circumstances of a residents’ death [39]. Such investigations bear the potential to reveal that staff’s unsafe care provision contributed to the resident’s death. If they do, the regulator (the Care Quality Commission) has a duty to prosecute the staff [40]. As a result, senior staff ensured that GPs visited residents with ICDs periodically or ad-hoc to avoid a Coroner’s referral and enable the certifying GPs to release the MCCD confidently and without reporting neglect because familiar with the resident’s deterioration and care.

### Senior Staff’s Use of Anticipatory Prescribing, Storing and Reviewing

Senior staff used the process of ICDs prescribing, storing, and reviewing to: (1) provide residents access to pharmacological symptom control on-site; (2) identify residents’ ceiling and location of care as non-emergency care in the care home; and (3) demonstrate safe care provision to the GP certifying the medical cause of death. ICDs thus performed a medico-legal function that helped senior staff manage residents’ uncertain dying trajectories within the care home setting and prevented most hospital admissions at the end-of-life.

#### Providing access to pharmacological symptom control

Senior staff prioritised mechanisms that ensured dying in the care home wherever possible, believing that care homes provided a more comfortable EOLC for both the resident and their visitors. One of these mechanisms was that, in senior staff’s experience, ICDs made hospital transfer unnecessary if residents experienced symptoms when dying because they provided access to symptom control on-site:

*“[Senior carer 8] insists that if anticipatory medications [ICDs] are not in place carers have no other options but to call an ambulance to make distressed residents comfortable, and paramedics take residents to hospital 9 times out of 10. While when ICDs are present, carers will call the district nurses to administer ICDs and make distressed residents comfortable.” [Fieldnote extracts 13:277 − 78]*.

ICDs allowed for adequate symptom control in most cases according to senior staff (Table 5). However, when ICDs failed to control dying symptoms, senior staff did not seek emergency care for the resident, but escalated healthcare provision by contacting palliative care services (Table 5). Further, senior staff reported that the level of symptom control provided by ICDs was often unnecessary, therefore ICDs were often not administered (Table 5). This indicates that pharmacological symptom control was not the only function of ICDs prescribing used by senior staff to prevent hospital transfers at the end-of-life.

**Table 5:** Care home staff’s experiences of ICDs administration

Staff experience	Example
ICDs are routinely prescribed (regardless of expected symptoms) but not routinely administered to residents.	*“it’s knowing the symptoms of when to use your Just In Case [ICDs] and when not to use it. We’ve got people in terrible pain […] there are people with no symptoms at all, up to the very last day […] Then we don’t use it. There’s no trend [concerning whether and when to administer ICDs], but the trend is that when the GP says that this person is end-of-life, then this paperwork [prescription, administration chart] and this medication [ICDs] are always in place. And that’s our practice.” [Nurse 5]*
Most residents die without needing ICDs administered but receiving other types of analgesia.	*“a lot of the time they [ICDs] are not needed, but we still have them and then occasionally you do get people [who need them] but most people are already on transdermal analgesia anyway” [Manager 4, who was also a registered nurse]*
ICDs are mostly effective in addressing residents’ symptoms	*“I’ve never been in the situation when I went to the maximum [dose of ICDs] and the resident was still in pain, or something. […] yeah, usually the maximum prescribed is perfect.” [Nurse 4]*
On the rare occurrence that ICDs are ineffective in addressing a resident’s symptoms, senior staff do not seek hospital admission for the resident.	*“she was agitated all the time so the district nurse called in [the local hospice team], they ended up coming in about three or four times over a week [before the resident died in the care home]. You can get some who just won’t settle, no matter what drugs you give them, we can’t get on top of the agitation, so they’re thrashing around all the time, that can be difficult for staff who aren’t used to it” [Manager 1]*

#### Identifying non-emergency care in the care home as the ceiling of care

Senior staff employed ICDs as a method to identify residents who were unsuitable for hospital transfer, as this nurse revealed:

*“I asked [nurse 8] what she meant by palliative residents. She replied that residents have end-of-life medication [ICDs] in place and are not for invasive treatment or hospitalisation, so their death is expected any time.” [Fieldnote extract 19:216].*

DT observed that residents with stored ICDs tended not to be escalated to emergency care, except in case of a traumatic fall or an accident. When the health of residents with ICDs deteriorated, senior staff instead accelerated healthcare provision by contacting the GP, as the case of resident Elaine exemplified:

*“After the GP prescribed end-of-life medications [ICDs], [Elaine] got used to have bouts of vomiting blood and being poorly every few months or so. The first time this happened [nurse 4] called in the GP to visit [Elaine]. The GP advised [Elaine]’s son that [Elaine] was not for investigation. They agreed to keep [Elaine] in the care home and the medications [ICDs] in place. Therefore, the care home nurses do not call an ambulance to send [Elaine] to hospital when she vomits blood but call the GP instead. Once [Elaine] was so unwell that [nurse 4] gave her a morphine injection. This was over a year ago now! [Elaine] is stable now but anything could tip her over. The other week the GP reviewed [Elaine’s] end-of-life medications [ICDs] at [nurse 4’s] request.” [Fieldnote extracts 13:62–71]*.

When ICDs were on site, GPs would typically advise the resident’s family and senior staff against hospital transfer, not only for potentially life-extending emergency care, but also for the planned investigation of unresolved symptoms. By contrast, when prescribed ICDs were not available on site, the norm was for senior staff to provide access to hospital care through emergency services for residents experiencing health issues which could not be addressed in the care home, as nurse Rachel example illustrates:

*“[Nurse 9] confided me that it is not easy to make end-of-life decisions. Once she had a frail resident who was bleeding from his catheter continually. [Nurse 9] kept calling the paramedics to send him to hospital: ‘The hospital kept phoning in to ask me why I was sending him, and I kept saying that it was my duty of care’. In the end, [Nurse 9] asked the GP to come in, prescribe end-of-life medication [ICDs] and discuss it with the family because the hospital did not want to take the resident anymore.” [Fieldnote extract 17:206]*.

The prescription, on-site storage, and GP review of ICDs thus acted as a clinical marker for senior staff to ascertain the location and ceiling of appropriate medical care for a resident as generalist provision in the care home. This allowed senior staff to rule out the provision of emergency care, which would often lead to a hospital transfer.

#### Demonstrating safe care provision

The on-site availability of ICDs allowed senior staff to withhold emergency care to residents experiencing an acute health crisis because it allowed them to document and demonstrate safe care provision if the resident died. Safe care provision involved the legal duty to provide (or provide access) to medical care preventing avoidable harm to residents [40]. Within this context, senior staff had a heightened awareness of the concept of neglect and their accountability, as this nurse conveyed:

*“I don’t want a sudden death here because if it’s something that you could have prevented and you haven’t, then that’s down for neglect, isn’t it?” [Nurse 1]*.

When a resident died in the care home, senior staff was accountable for the demonstration of safe care to the GP certifying the medical cause of death. A GP’s suspicion of neglect or refusal to release MCCD triggered a Coroner’s investigation [36]. If the Coroner’s investigation revealed unsafe care provision, the regulator (the Care Quality Commission) had a duty to prosecute the staff [40]. Crucially, the prescription, storing and GP review of ICDs minimised the likelihood of a Coroner’s referral and investigation when a resident died in the care home.

First, DT observed that senior staff considered medical records and GPs’ notes in residents’ care plans to constitute documentary evidence of safe care provision. The storing of ICDs prescriptions and administration charts, and GPs keeping a log of ICD reviews in residents’ care plans allowed senior staff to evidence to the GP releasing the MCCD that the resident’s death was expected, and adequate care had been provided and planned. This enabled different GPs from the GP practice allocated to the care home to release the MCCD confidently and without raising suspicions of neglect.

Second, storing prescribed ICDs on-site allowed senior staff to involve the GPs in a resident’s EOLC by instigating periodic or ad-hoc GP visits to review the ICDs (Sect. 3.1.2). This maximised the likelihood to: (1) meet the 14 days period before death which avoided an automatic Coroner’s referral; and (2) have the certifying GP release the MCCD confidently and without raising suspicions of neglect, thereby avoiding an in-depth Coroner’s investigation (Sect. 3.1.2). This enabled most senior staff to feel *“protected”* when deciding to withhold emergency care to a dying resident and facilitate a care home death *[nurse 8 from fieldnote extracts 19:93].*

By contrast, on the rare occasion that a GP had not reviewed the ICDs in many months (about 5+) and the resident experienced a sudden life-threatening health crisis, some senior staff did not feel confident to withhold emergency care, as nurse Olivia confided me:

*“For [nurse 10] ‘if you are in doubt, you always need to call an ambulance, you need to cover your back, otherwise you are in trouble’. If residents with end-of-life medications [ICDs] have a seizure or heart attack and they have not been seen by a GP in five or six months or more, she will call an ambulance because the residents were stable before and, if they die, she cannot justify not calling an ambulance to the GP.” [Fieldnote extracts 19:20–21]*.

This counterexample confirms the interaction between the clinical and legal functions of ICDs prescribing. When senior staff doubted the certifying GP to agree with their clinical decision to forego emergency care, because not supported by a recent GP review of ICDs, they felt exposed to the legal consequences of a GP Coroner’s referral for neglect or refusal to release the MCCD. This led some senior staff to provide emergency care to acutely ill residents whose ICDs had not been reviewed in about five months or more, triggering a hospital transfer at the end-of-life.

## Discussion

### Main findings

Senior staff use the process of ICDs prescribing, storing, and reviewing as a mechanism to manage residents’ uncertain dying trajectories on the care home’s premises and avoid hospital transfer at the end-of-life. Senior staff described how GPs usually welcome senior staff’s requests to prescribe ICDs and review prescribed ICDs for as long as residents survive. In the absence of clinical guidelines specifying a timeframe for ICD review, senior staff seek to match ICD review to the period before death which prevents an automatic Coroner’s referral (14 days pre-pandemic, currently 28 days). Senior staff invite GPs to review ICDs to verify and document the clinical appropriateness to: (1) administer ICDs if residents are symptomatic, relying on care home or district nurses’ assessment; and (2) withhold potentially life-extending emergency care weeks, months or even years after a GP first prescribed the ICDs. In turn, a GP review of ICDs enables senior staff to (3) demonstrate that the resident’s death was expected, and adequate care had been provided and planned to the GP certifying the medical cause of death. This minimises the likelihood of a Coroner’s investigation and criminal prosecution by the regulator for unsafe care provision. Senior staff thus use the GP prescription and review of ICDs as a medico-legal system to identify and support a resident’s EOLC and death in the care home as a clinically and legally appropriate outcome. As a result, ICDs medico-legal function enables senior staff to prevent most residents’ hospital transfers at the end-of-life which, in their experience, follow from seeking emergency ambulance care.

### What this study adds

This study provides new and significant insight into senior staff’s role and use of the process of anticipatory prescribing in care homes. Previous studies evidence that ICDs prescribing reduces residents’ hospital death and admission in the last month of life [17, 18]. However, these studies under-estimate senior staff’s role in realising this outcome and their use of ICDs prescribing beyond symptom control. This study adds that senior staff interpret and use ICDs as the mechanism through which the GP’s medico-legal authority to make life-altering EOLC decisions is extended *to them*.

Senior staff want to provide clinically appropriate care to the residents. However, what clinically appropriate care is, is not always clear concerning emergency care provision at the end-of-life. Many residents survive one or more health crises from which both senior staff and GPs expected them to die. These extended and unpredictable dying trajectories are typical of care home residents [22, 25, 28, 41], who are more likely than older adults receiving care at home or in a hospice to die with dementia and extremely low activity levels [42]. Nonetheless, they expose senior staff to prognostic uncertainty and the possibility that the certifying GP refers the death to the Coroner for suspected neglect and/or inability to release the medical certificate of cause of death (MCCD) confidently.

To manage residents’ uncertain dying trajectories and avoid the legal risk of a Coroner’s investigation, senior staff ask GPs to prescribe ICDs weeks ahead of a resident’s expected death and to *not* de-prescribe ICDs for as long the resident survives. This mirrors approaches to ICDs prescribing and storing in home and residential care home settings, where district nurses advise GPs on the timing of ICD prescription to home-dwelling patients [19] and ICDs are stored in patients’ homes or residential care homes for up to three years [43]. It also allows senior staff to trigger ad-hoc or periodic GP reviews of ICDs, thereby performing two medico-legal functions simultaneously. First, a GP review of ICDs allows senior staff to verify that supportive care in the care home and, if needed, ICDs administration, constitute clinically appropriate care provision when the resident experiences life-threatening symptoms. Second, a GP review of ICDs reduces the likelihood that the certifying GP refers the death to the Coroner for suspected neglect and/or unfamiliarity with the resident’s deterioration and care. Senior staff consider these medico-legal functions to enable them to support residents to die in the care home, thereby avoiding emergency hospital transfers at the end-of-life.

### Strengths and limitations

Care home senior staff’s use of ICDs prescribing is a relevant and under-researched area in community EOLC practice. The observational and inductive approach to data collection is novel in this area and limits confirmation bias with respect to the hypothesis that senior staff use ICD prescribing to reduce the likelihood of residents’ transfers to hospital. Further, the triangulation of observational and interview data, and the inclusion of a relatively large number and diverse type of care homes, strengthens the validity and transferability of the findings. The main limitations of this study are the lack of engagement with GPs’, residents’, and relatives’ viewpoints about senior staff’s use of ICDs prescribing. Future research should investigate the acceptability of ICDs prescribing to residents and relatives and whether it influences residents’ care beyond hospital transfer avoidance. This study is also limited to the English context. Future research should include all British nations and other countries where anticipatory prescribing is practiced.

## Conclusions and recommendations

This study reveals that senior staff pursue the anticipatory prescribing, on-site storing and GP review of ICDs as a medico-legal system to enable them to support residents to die in the care home as opposed to in hospital. GPs’ prescriptions and reviews of ICDs thus perform a similar function to emergency care plans written by clinicians and advising against hospital transfer [44, 45]. However, senior staff experience difficulties interpreting such clinician-led plans [44], at times leading to emergency care provision and hospital transfer at the end-of-life [28, 44]. By contrast, senior staff interpret and pursue a GP’s prescription or review of ICDs as a clear clinical indication that the resident is for supportive care in the care home as opposed to emergency care. ICDs thus appear to be more effective than clinician-led emergency care plans in preventing that dying residents are transferred to hospital. However, ICDs and emergency care plans have different safety [46], acceptability [20, 43] and cost [47] implications that future research should explore.

Ambiguity about whether ICDs identify a resident as unsuitable for emergency care arises only when a GP has not reviewed the ICDs in about five months or more. A clear timeframe for the review of ICDs would avoid this ambiguity and create a strong framework for EOLC in care homes. It is recommended here that the timeframe should match the period before death in which a doctor must visit a patient dying in the community to release the MCCD without triggering an automatic Coroner’s referral. This period is currently 28 days [37] (was 14 days pre-pandemic) [38]. Such specificity would promote coordination between senior staff and GPs, avoid unnecessary Coroner’s referrals, and make residents’ dying in care homes less reliant on personal relationships of trust and the individual skills of senior staff in predicting dying.

Finally, this study highlights that GPs’ prescription and review of ICDs is often a clinical decision for which relatives’ consent (and rarely residents’ consent) is gained rather than sought. Whilst it may be presumed that hospital transfer may not benefit very frail and/or dying residents, some residents or relatives might want to prioritise potentially life-extending healthcare provision until the last hours of life or prefer a hospital death to a care home death. It is therefore important to reflect on the multiple roles of anticipatory prescribing in the care home sector which this paper revealed.

## Data Availability

The anonymised interview data used in this study may be requested by researchers through contacting the lead author (DT). Given the sensitive subject nature of the anonymised qualitative data, they may be made available to researchers with evidence of a study protocol and Research Ethics Committee approval, and on completion of a data use and sharing agreement.

## Abbreviations

EOLC: End of life care
GP: General practitioner
ICD: Injectable controlled drug
MCCD: Medical certificate of cause of death
